# Isolated metastasis of the bowel from undifferentiated thyroid carcinoma. A case report and a literature review

**DOI:** 10.1007/s00384-025-04804-1

**Published:** 2025-01-22

**Authors:** Mariacristina Cartillone, Iacopo Sarvà, Chiara Mazzone, Giorgia Litrico, Maria Carolina Picardo, Francesco Saverio Latteri, Gaetano La Greca

**Affiliations:** General Surgery, Cannizzaro Hospital, Catania, Italy

**Keywords:** Thyroid carcinoma, Metastasis thyroid, Metastasis bowel, Thyroid tumor, Intestinal perforation

## Abstract

In this article, we aim to demonstrate that thyroid carcinoma can metastasize to the small bowel. This case report involves a 66-year-old woman who underwent total thyroidectomy surgery in 2019, with histopathology revealing a 3A undifferentiated thyroid cancer. She presented with symptoms of bowel obstruction, including abdominal pain, nausea, and vomiting. Thyroid carcinoma accounts for less than 4% of all malignant neoplasms, making it the most common endocrine malignancy. The predominant type is papillary carcinoma, which generally has a favorable prognosis. In contrast, poorly differentiated thyroid cancers have a poor prognosis, with a 5-year postoperative survival rate of 66%. Common metastatic sites include the lungs, brain, and bones, with rare occurrences in organs such as the kidneys, spleen, adrenal glands, and ovaries. Intestinal metastases are extremely rare, with fewer than 15 cases of gastrointestinal localization documented in the literature. This case highlights the rare possibility of thyroid carcinoma metastasizing to the small bowel, emphasizing the need for clinicians to consider metastatic spread in patients with a history of thyroid cancer who present with gastrointestinal symptoms.

## Introduction

Thyroid cancer is the most common endocrine malignancy, representing 3.4% of all malignant cancers [1]. More than 95% of thyroid cancer cases originate from follicular epithelial cells, while 5% arise from the parafollicular C cell (medullary thyroid cancer). Follicular thyroid cancer affects more often women (75%) aged between 40 and 60 years old [2], and leads to death in 18% of cases at 10 years, in 28% of cases at 20 years, and in 40% of cases at 30 years [2]. Well-differentiated thyroid tumors usually metastasize locally and involve the cervical lymph nodes; in 20–31% of cases, this type of metastasis can be found at the time of the first diagnosis [3]. Our experience talks about a 66-year-old woman who has metastatic intrabdominal localization resulting from undifferentiated thyroid carcinoma. This tumor and its metastasis were insidious and very aggressive and led the woman to death. The symptoms were aspecific.

Literature has described only 5–10% of patients with PTC present metastases at first diagnosis: over 50% have only lung involvement, 25% bone metastases, 20% have both lung and bone involvement, and about 5% develop distant metastases in other sites [4]. On the contrary, poorly differentiated thyroid cancers are more aggressive with a tendency to vascular invasion and metastasize hematogenously to distant sites, in particular to lungs, brain, and bones [5]. More than half of patients at the time of diagnosis present already distant metastases [4], with a mortality rate of 50% at 10 years [2]. Intrabdominal metastases are very rare. Less than 15 cases of gastrointestinal metastases thyroid carcinoma have been described in the literature.

## Case report

**Fig. 1:**
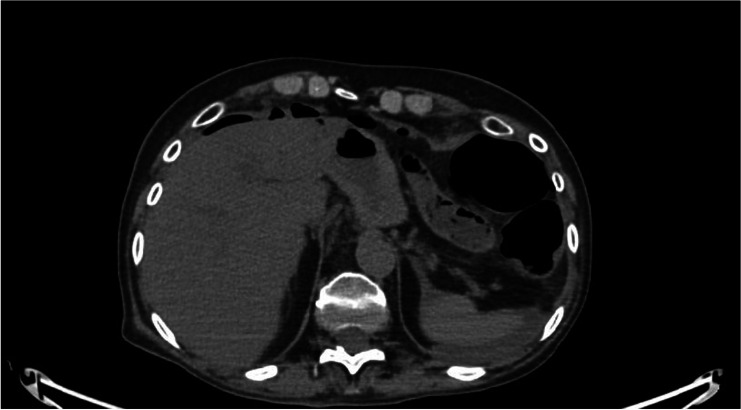
Meteoric overextension of the colon

**Fig. 2:**
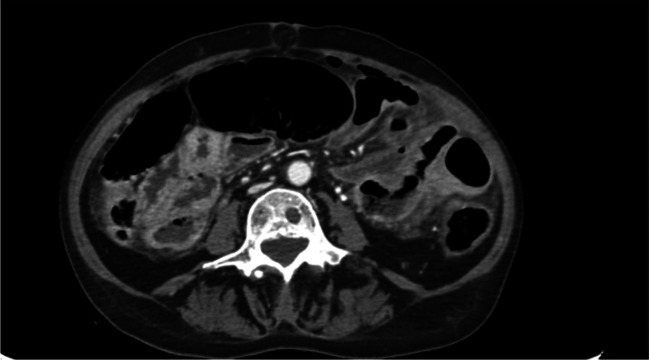
Supramesocolic and submesocolic fluid level

A 66-year-old Caucasian woman presented to the Surgical Department with acute pain in the upper quadrants of her abdomen, bowel obstruction, nausea, and vomiting. The patient manifested oliguria, tachypnea, and tachycardia (160 bpm) without fever. The blood pressure was 130/80 mmHg. Her medical history reported undifferentiated thyroid cancer (she underwent total thyroidectomy surgery in 2019, with histopathology revealing a 3A thyroid cancer) with metastases in the lung, brain, and liver. She was under radiotherapy treatment. Laboratory tests showed high levels of inflammatory markers (PCT 117 ng/ml, WBC 1.1 × 10^9^/l, PCR 35 mg/l). Abdominal and chest CT scans showed the presence of pneumoperitoneum with a distal ileus tract with thickened walls, suggesting an intestinal perforation (Figs. 1 and 2).

## First surgical intervention

Surgery was scheduled: a supraumbelicopubic midline incision was made, confirming the perforation of the last ileal loop, caused by an intraluminal mass. A resection was performed with a ileo-ileal antiperistaltic latero-lateral mechanical anastomosis. In the immediate post-operatory, the patient was transferred to the intensive care unit. The tissues seemed strong enough to withstand this surgical choice, so we adopted for an anastomotic technique to allow an easier living condition rather than having a stoma and because having an ileostomy can cause a significant hydroelectrolytic imbalance.

A gradual improvement of her clinical conditions (oriented, cooperative, stool and gas intestinal movement) and the hemodynamic values allowed the transfer to the Department of General Surgery on the 4th postoperative day. On the 12th postoperative day, the patient suffered from abdominal pain with a worsening of the blood laboratory tests (PCR 2.1 mg/l; WBC 12.9 × 10^9^/l; neutrophilic cells 87.2%; platelets 100 × 10^9^/l); moreover, a biliary output from the drain appeared.

## Second surgical intervention

An exploratory laparotomy was performed, revealing a blood and biliary collection in the abdominal cavity caused by an anastomotic dehiscence. Resection of the anastomosed loop and a terminal ileostomy were then performed. She died on the 14th postoperative day. Histological examination revealed the presence of a poorly differentiated neoplasm, with the presence of findings compatible with cancer localization from a previous thyroid tumor.

## Discussion

The most prevalent type of thyroid cancer is papillary thyroid cancer, which occurs in follicular epithelial cells. It has an excellent prognosis, with a 10-year survival rate of 42%. This type of cancer typically affects women more often than men and tends to spread locally, sometimes leading to metastasis in cervical lymph nodes.

Poorly differentiated thyroid cancer and anaplastic thyroid cancer are less frequent, both with a poor prognosis and a higher tendency to metastasize to distant sites, especially lungs, bones, and brain.

Distant metastases occur in less than 10% of patients with papillary and follicular thyroid carcinoma and represent the most frequent cause of thyroid cancer-related death [6], with an overall survival rate of 42% at 10 years, and less than 30% at 20 years [7]. In literature, there are only a few cases of metastasis of the abdominal organs from thyroid cancer; in most of these cases, the organ affected by the metastasis is the colon. We searched for their attitude and their consequences.

Only one case of adrenal gland metastasis from Hurtle cell tumor can be found, involving a 76-year-old woman who underwent hemithyroidectomy 10 years earlier [8].

Another infrequent site of metastasis from thyroid cancer is the kidneys; usually, these patients experience symptoms such as hematuria, abdominal pain, or a sense of abdominal weight [9].

A case report describes two cases of renal metastases, both deriving from the papillary follicular variant of thyroid cancer and both involving two 60-year-old women, asymptomatic and with normal blood tests; only with CT was it possible to detect the renal mass.

Regarding splenic metastases from thyroid cancer, these are not frequent. One case report described spleen involvement from poorly differentiated thyroid cancer in a 75-year-old woman [10].

Three cases of ovarian metastases resulting from papillary thyroid cancer were found in 35-year-old women [11–13]. Gastrointestinal metastases are infrequent, accounting for 0.5–1% of all distant metastases [7]. In the literature, just a few cases are described as shown in Table 1.

**Table 1:** Case reports of intrabdominal localization thyroid metastasis cancer

Authors	Year	Age	Sex	Location		Prognosis
Bandorski D. [14]	2002	75	M	Duodenum and jejunum	1 year after thyroidectomy	Death after 8 days after surgery for respiratory and circulatory insufficiency
Meyer G. Y. [15]	2009	28	F	Bowel	3 years after surgery	Death few months after surgery. She didn’t respond to radioiodine therapy
Kobayashi M. [16]	2015	60	M	Small intestine and thoracic esophagus	2 years and 10 months after surgery	Death 9 days after surgery for dyspnea caused by tracheal stenosis
Hosoda K. [17]	2020	74	M	Colon	6 years after thyroidectomy	Death 1 month after surgery
Hrudka J. [18]	2021	66	F	Small bowel	2 months after thyroidectomy	Death after orotracheal intubation. No operation
Ricciardelli [19]	2006	unknown	unknown	Small bowel	5 months after thyroidectomy	Death 1 week after surgery

In some cases, the onset of the pathology can appear after years, as in our case, other times the onset can be earlier.

In one case report, a patient underwent left hemithyroidectomy and right hemithyroidectomy 1 year apart. Two weeks after right hemithyroidectomy, the patient began to experience abdominal pain, anorexia, weight loss, nausea, and vomiting [20].

In our case, the pathology manifested itself about 4 years after the thyroidectomy.

Clinical presentations of small intestinal metastasis can vary from bowel obstruction, gastrointestinal bleeding, or perforation.

Klubo et al. [21] reported two case studies. In the first case, the patient presented with an esophageal mass that was detected through an endoscopy after an episode of hematemesis. The second case was a patient with colon cancer derived from PTC, who had already undergone surgical treatment and whose symptoms began with rectal bleeding. In our case, the patient showed signs and symptoms related to their gastrointestinal tract, such as abdominal pain, a lack of bowel movements, nausea, and vomiting, which led to an occlusive syndrome.

In a case report, Hosoda et al. reported that the first signs of colon tumor were renal dysfunction, elevated white blood cell count, and C-reactive protein levels [17].

In a case study, Richards et al. reported aggressive metastasis of thyroid cancer to rectum, with rectal bleeding that required a total procto-colectomy with an abdominal-perineal resection and a permanent ileostomy [22].

As reported in the literature, metastases in thyroid cancer are frequently associated with poor prognosis, often leading to patient mortality. However, no established guidelines currently exist to inform tailored treatment strategies for these cases. In the present case, the implementation of a multidisciplinary approach would have been beneficial. Unfortunately, due to the emergency nature of the patient’s presentation, it was not feasible to organize such a meeting.

## Conclusion

Thyroid cancer mostly impacts women in the age range of 40 to 60 years. Differentiated carcinoma typically has a more favorable outlook compared to undifferentiated thyroid cancer. For differentiated thyroid cancer, the rate of distant metastasis upon initial diagnosis is approximately 4%. ATC typically has a sudden onset and rapid progression. The most frequent sites of metastasis include the lung (25%), mediastinum (25%), liver (10%), bone (6%), kidney/adrenals (5%), heart (5%), and brain (3%) [23].

Based on the literature, it is more common for women in their 6–7th decade of life to be affected by thyroid cancer metastases at certain sites. Metastasis from thyroid cancer rarely occurs at these sites, but they are very aggressive and insidious. The patients may not show symptoms or may have nonspecific symptoms, and their blood tests are usually normal. Our case report emphasizes that undifferentiated thyroid cancer can spread to the abdomen and gastrointestinal system with a poor prognosis. Abdominal pain or gastrointestinal bleeding in patients with thyroid cancer needs to be investigated for suspected distant metastases. CT scans are essential for accurate diagnosis and timely intervention. Complete surgical resection is the only palliative treatment option. Evaluating the presence of previous thyroid cancer can help us choose the surgical technique to adopt. So the choice to do the intervention surgery must be the first choice. But given the aggressiveness of this tumor even at a distance, perhaps it is better to adopt a more conservative approach with the creation of an ileostomy rather than attempting an emergency anastomosis given the weakness of the tissue.

## Data Availability

No datasets were generated or analysed during the current study.
